# Seventh District Dental Society of Ohio

**Published:** 1892-06

**Authors:** 


### PROCEEDINGS.

### Seventh District Dental Society of Ohio.

The annual meeting of this society was held on Tuesday, May 17th, at Washington Court House.

The society was called to order at 10:30 A. M., and continued till 4 o'clock in the afternoon. President A. O. Heise in the chair; Dr. J. R. Callahan, Secretary. There were about twenty-five members present. Pres. Heise read his address, which was a consideration of the legal status of the profession in Ohio, expressing gratification for the amendments made to the law by the last legislature, and expressing the hope that it would receive the sanction and support of the profession throughout the State. The subject was discussed by several of the members, each of whom expressed appreciation and approval of the law. After this a paper was read by Dr. L. E. Custer, of Dayton, Ohio, subject: "The Influence of Tobacco upon the Teeth of Tobacco Chewers." The opinion presented in the paper was, that the influence of tobacco upon the teeth is not as injurious as it is usually supposed to be, neither did the paper affirm that tobacco in any sense is beneficial to the teeth. Quite a spirited discussion was had upon the paper, several of the members taking part; some assuming the position that the antiseptic property of tobacco was beneficial in a degree; others maintaining that there was but little, if any, influence of this kind in connection with its use; others expressed the opinion that the exercise given to the teeth by the act of mastication strengthened them, especially in their periosteal attachment; others taking exception, saying that such a result was only occasionally found, that in many instances the teeth of tobacco chewers instead of being strengthened in their attachment were weakened and became loose by absorption of the tissues, and the teeth oftentimes lost in this way; it was the general opinion expressed that the teeth of tobacco chewers suffered by mechanical abrasion, more than the teeth of the non-users. Instances were mentioned in which the teeth were worn off to the margin of the gums by this kind of attrition. The habit was regarded by all as a filthy, disgusting and useless one; at least, the user of the weed found very little, if any sympathy in the consideration of that paper.

After the conclusion of this discussion a recess of one hour was taken.

The meeting convened again at one o'clock, when Dr. Custer closed the discussion upon his paper, which consisted chiefly in emphasizing the points presented.

The election of officers for the ensuing year was now declared to be in order, and a ballot being had, Dr. B. F. Johnson, of Camden, Butler Co., was chosen President; J. F. Dennis, of Washington C. H., Vice President; J. R. Callahan, of Cincinnati, Secretary; I. W. Keeley, of Oxford, Treasurer. The Executive Committee: Drs. J. H. Sillito, of Xenia; L. E. Custer, of Dayton, and Chas. Welch of Wilmington.

Camden, Butler County, was chosen as the place of the next annual meeting; and it was decided to continue the meeting two days. It was also decided to extend an invitation to the Eastern Indiana Dental Society to hold a joint meeting at the place above indicated, and at a time that would be acceptable to both parties. The Executive Committee was given discretionary power to extend the invitation, to make the arrangements, and fix the time for the meeting.

Dr. Callahan gave notice that next year he would make a motion to defer all business to the Board of Directors, in order that the greatest amount of time could be secured for the prosecution of practical and scientific work during the meetings of the Body.

A paper on "Tumors of the Mouth" was read by J. Taft, and discussed by Drs. C. M. Wright, A. O. Heise, Otto Arnold, W. D. Tremper, and J. Taft.

Though the paper brought under consideration only simple, benign tumors of the mouth, yet other varieties of tumors were considered, and many good thoughts and valuable suggestions made.

After some further unimportant routine business, this Society adjourned to meet as above indicated. The meeting was a very pleasant one indeed, and in a social way was enjoyable to all present. Several were present who are not often seen at our meetings. Another encouraging feature was that most of those present took part in the discussions and otherwise, and some who very seldom, if at all, have been elsewhere heard. If the entire territory of our State could be embraced at such gatherings, as was contemplated in arranging the States in Districts, very great advantage would accrue, and many would be brought out, and, as in this case, take part that otherwise would rarely, if ever, be seen in such meetings; and we hope that a warm interest will in the future be manifested for the success of this Society.

